# Does Cannabidiol Protect Against Adverse Psychological Effects of THC?

**DOI:** 10.3389/fpsyt.2013.00130

**Published:** 2013-10-16

**Authors:** Raymond J. M. Niesink, Margriet W. van Laar

**Affiliations:** ^1^Trimbos Institute, Netherlands Institute of Mental Health and Addiction, Utrecht, Netherlands; ^2^Faculty of Natural Sciences, Open University of the Netherlands, Heerlen, Netherlands

**Keywords:** tetrahydrocannabinol, cannabidiol, cannabis, psychosis, anxiety, drug dependence, cognition

## Abstract

The recreational use of cannabis can have persistent adverse effects on mental health. Delta-9-tetrahydrocannabinol (THC) is the main psychoactive constituent of cannabis, and most, if not all, of the effects associated with the use of cannabis are caused by THC. Recent studies have suggested a possible protective effect of another cannabinoid, cannabidiol (CBD). A literature search was performed in the bibliographic databases PubMed, PsycINFO, and Web of Science using the keyword “cannabidiol.” After removing duplicate entries, 1295 unique titles remained. Based on the titles and abstracts, an initial selection was made. The reference lists of the publications identified in this manner were examined for additional references. Cannabis is not a safe drug. Depending on how often someone uses, the age of onset, the potency of the cannabis that is used and someone’s individual sensitivity, the recreational use of cannabis may cause permanent psychological disorders. Most recreational users will never be faced with such persistent mental illness, but in some individuals cannabis use leads to undesirable effects: cognitive impairment, anxiety, paranoia, and increased risks of developing chronic psychosis or drug addiction. Studies examining the protective effects of CBD have shown that CBD can counteract the negative effects of THC. However, the question remains of how the laboratory results translate to the types of cannabis that are encountered by real-world recreational users.

Tetrahydrocannabinol (THC) is the main psychoactive substance in cannabis. Cannabidiol (CBD) is a cannabinoid that appears in cannabis resin but rarely in herbal cannabis. In recent years, many positive attributes have been ascribed to CBD. Is cannabis that contains CBD less harmful than cannabis without CBD? Are people who smoke cannabis resin, therefore, less susceptible to psychosis or less likely to become addicted than are people who smoke herbal marijuana? In this article, several of the health aspects of CBD will be reviewed. The article will focus on the role played by CBD in contributing to the psychological effects that are experienced during recreational cannabis use.

## Pharmacology

*Cannabis sativa* contains more than 80 different cannabinoids, of which THC is principally responsible for the pharmacological actions, including the psychoactive effects. THC binds to specific proteins in the brain – the cannabinoid receptors (CB-Rs) (1). Two different receptors have been discovered: the CB1 and CB2 receptors (2, 3). CB1-R is mainly found in the central nervous system (CNS); CB2-R is predominantly present in the immune system (3–5). Endocannabinoids are naturally occurring substances that attach to these receptors (6–8).

Cannabinoid receptors, endocannabinoids, and the enzymes involved in the synthesis and degradation of these substances together form the endocannabinoid system (9). The activation of the CB-Rs affects the actions of various neurotransmitters, such as acetylcholine, dopamine, GABA, glutamate, serotonin, norepinephrine, and endogenous opioids (10, 11). Under normal physiological circumstances, CB-Rs are activated by endocannabinoids (12). The activation of CB-Rs by endocannabinoids inhibits excessive neurotransmitter release. Endocannabinoids are lipid-soluble compounds, which prevent them from traveling long distances within the brain. As a consequence of this feature, endocannabinoids are ideally suited for small-scale, local physiological processes (13).

Tetrahydrocannabinol mimics the effect of endocannabinoids. In contrast to these substances, THC is not rapidly broken down at the site of operation, and it not only works at specific locations but simultaneously activates all CB receptors throughout the brain (14).

The mechanisms by which CBD exerts its effect are not precisely known, but it is clear that the pharmacological actions of CBD follow from many different mechanisms [for reviews, see Ref. (15, 16)]. CBD weakly binds to CB-Rs but is capable of antagonizing the effects of THC, even when the former is present in low doses. By inhibiting the degradation of the endogenous cannabinoid anandamide, CBD intensifies, and prolongs its effect (17). The (extended) presence of anandamide prevents THC from interacting with CB-Rs. CBD also interacts with several other recently discovered CB-Rs, and it is an agonist for the 5-HT_1A_ receptor (18, 19), which may explain some of the antipsychotic and anxiolytic effects of CBD (20). Through its effect on intracellular calcium concentrations, CBD might protect neurons against the possible neurotoxic effects of THC (21). CBD itself has almost no effect on normal physiological processes. Only when a stimulus (such as pain or a shock reaction) or another cannabinoid (such as THC) upsets the normal “tone” of the endocannabinoid system is the effect of CBD expressed (12).

The amount of CBD administered, the ratio of CBD to THC and the timing of administration all seem to be important in determining the possible effects of CBD (22, 23). Most clinical studies on the effects of CBD are not relevant for generalizing to the effects of CBD in “recreational” cannabis users. In many of these studies, the doses that have been used are not relevant to the situation typically encountered by recreational cannabis users.

Clinical research has focused on the physical effects of cannabis use, such as pain relief, appetite promotion, and inflammation. For recreational cannabis users, the substance’s psychological effects are the most important. In many experimental studies, the routes of administration used for both THC and CBD are not comparable to the routes of administration found in recreational cannabis use. The high dosages of CBD that have been used in experimental studies increase the concentration of CBD in the blood to levels that can never be reached by smoking a joint. The method that is most comparable to smoking is exposure through a vaporizer, but little research has been conducted involving the administration of cannabis, THC, or CBD via a vaporizer (24, 25). Therefore, it is unknown to what extent the effects of a single administration procedure can be extrapolated to recreational cannabis users given such differences in usage patterns.

## Toxicology of CBD

Research on the pharmacological and toxicological properties of CBD has been performed on different types of animals. In general, the metabolism of CBD in different species seems similar to that observed in humans, but some differences exist (26). It is possible that differences in metabolism and kinetics among different species have been responsible for some of the observed differences in pharmacological and toxicological effects.

Little research has focused on the safety and side effects of CBD in humans. However, several studies have described the effects of CBD for therapeutic applications in clinical trials. Only a few, generally mild side effects have been observed after administration of CBD in these human studies, though a wide range of effects over a wide dose range, including acute and chronic administration, have been examined. Few undesirable effects are reported, and tolerance for CBD does not seem to occur.

Based on an extensive literature review, Bergamaschi and colleagues concluded that CBD, to the extent that it has been studied, is a substance with low toxicity (27). Notably, however, the absence of harmful effects of CBD in humans has been described in research that was not primarily aimed at investigating these same side effects or toxicities of CBD. Because no specific research on these issues has been performed, it is currently impossible to draw conclusions about differences in toxicity between hashish and marijuana.

Chronic cannabis use is associated with psychiatric toxicity and cannabis has been implicated in the etiology of long-term psychiatric conditions (28). Several *in vivo* brain scanning techniques have been conducted to investigate whether chronic, heavy cannabis use leads to structural changes in the brain [for reviews, see Ref. (29, 30)]. The results of these studies have been relatively inconsistent. In general, no differences in total brain volume between cannabis users and non-users have been found. With respect to CB1 receptor concentrations in different parts of the brain, it can be expected that structural changes after chronic intensive cannabis use would most likely eventually be situated in the orbitofrontal cortex (OCC), the anterior cingulate cortex (ACC), the striatum, the amygdala, and the hippocampus (31–33). In some structural magnetic resonance imaging (sMRI) studies, reductions in the volumes of the hippocampus, the amygdala, and the cerebellum have been found in adult heavy cannabis users when compared with healthy controls (21, 34, 35). Using a PET scan technique, Wilson and colleagues found age-dependent morphological changes in early-onset cannabis users. In subjects who started their cannabis use before the age of 17, it has been found that the ratio of cortical gray to white matter is smaller when compared with subjects who had started using cannabis after their 17th birthdays (36). Structural abnormalities due to chronic cannabis use have been most consistently identified in the hippocampus (21, 34, 35). Using a voxel-based morphometry (VBM) approach, Demirakca and colleagues studied gray matter (GM) concentrations and volumes of the hippocampus in 11 chronic recreational cannabis users and 13 healthy controls and correlated their findings with THC and CBD measurements made from hair analyses. They found that cannabis users showed lower GM volume in the right anterior hippocampus. Higher THC and lower CBD were associated with this hippocampal volume reduction, suggesting neurotoxic effects of THC and neuroprotective effects of CBD.

The conflicting results among volumetric brain studies seem to result from differences in time span (e.g., age of onset), patterns of cannabis use (e.g., frequency, duration of use, cumulative lifetime use), and type of cannabis used (e.g., potency, CBD/THC ratio) (29, 30).

## Psychological Effects

The effects of cannabis on psychological functioning mainly concern psychotic symptoms, anxiety, depression, cognitive functioning, and the potential for abuse and dependency. Several studies show that high doses of cannabis can provoke acute and transient psychotic reactions in both “healthy” users and in people with a certain predisposition for psychosis (37–39). These effects are dose-related (i.e., more THC produces a greater effect) and are stronger and longer-lasting in naive and occasional users than they are in frequent and transient cannabis users. Rottanburg and colleagues were the first to propose a protective effect of CBD on THC-induced psychosis. They suggested that the high incidence of cannabis-related psychosis among their patients occurred because cannabis variants in South Africa are more potent in terms of THC content and because they lack CBD (40).

As early as 1982, there were indications that the psychosis- and anxiety-inducing effects of THC can be suppressed by CBD (41, 42). Several other studies have found support for the antipsychotic effects of CBD. fMRI studies have shown that the effects of THC are correlated with a decrease in brain activity in the striatum. The striatum plays an important role in planning activities, modulating motor activity (movement), and performing cognitive tasks. CBD has been found to increase the activity in this brain area (43). Moreover, in other brain areas, the effects of CBD on neurological activity have been shown to be opposite those of THC.

In one Dutch and three English studies, associations between the consumption of certain types of cannabis and the occurrence of psychotic symptoms were reported (41–47). The results of these “naturalistic” studies suggest that CBD exerts a dampening effect on THC-induced psychotic symptoms. It is not clear for which CBD/THC ratio and for what minimum CBD concentration the protective effects of CBD may be expressed. The main features of these “naturalistic” studies are summarized in Table 1.

**Table 1:** **Summary of “naturalistic” studies in which the effects of cannabidiol and cannabis with a high dosis of THC on psychological functions have been investigated**.

Reference	Subjects	THC/CBD	Results	Remarks
Di Forti et al. (47)	“First-episode” psychiatric patients (*n* = 280)	Self reported frequency and type of cannabis used	The chance that high-potent cannabis (THC) has been used is higher among “first-episode” psychotic patients than among non-psychotics	Also more frequent use in “first-episode” psychotic patients
Morgan and Curran (45)	Cannabis users (*n* = 154)	Grouping based on presence of THC and/or CBD in hair	More psychotic symptoms among THC group in comparison with no THC group and in group with THC and CBD in hair	THC might be psychotogenic and CBD might protect against this effect
Schubart et al. (48)	Websurvey among cannabis users (*n* = 1877)	Grouping based on self reported preference for type of cannabis	Less psychotic symptoms in cannabis users who use cannabis with high level of CBD (hash)	Personal communication with author (Schubart)
Morgan et al. (46)	Cannabis users, at least once a month (*n* = 134)	Choosing cannabis by cannabis user	Acute effects on mood, psychotic symptoms, and cognition	CBD attenuates the THC-induced memory impairment; CBD does not affect psychotomimetic symptoms
Morgan et al. (49)	Recreational cannabis users (*n* = 54) versus daily users (*n* = 66)	Measuring THC and CBD in hair	THC increases possibility of negative psychotic symptoms, CBD antagonizes (part of) THC-induced effects	

Longitudinal studies that have investigated the relationship between chronic cannabis use and the occurrence of psychosis have shown that cannabis use increases the risk of later psychotic symptoms and disorders by a factor of 2–3. The magnitude of the risk depends on the degree of exposure, the age of onset of cannabis use and the “vulnerability” of the user (50–52). No longitudinal studies have distinguished between the type of cannabis having been used, and no studies give an indication of the THC/CBD ratio.

One case-control study has shown an association between the occurrence of a first psychotic episode and the use of high-potency cannabis (skunk or sinsemilla) (47). Patients with psychotic symptoms had more frequently used skunk or sinsemilla cannabis instead of hashish than had non-patients. Patients experiencing first-episode psychosis were also more likely to be daily users of high-potency cannabis than were controls. This finding suggests that both the daily use and consumption of cannabis with a high-THC and low-CBD content increase the risk of developing psychosis.

Cannabis use can lower the age of a first psychotic episode (53, 54). Epidemiological and clinical studies suggest an adverse effect of cannabis use on the course of the disease in terms of relapse, exacerbation of symptoms and number of hospitalizations (38, 55–57). With the exception of a study by Di Forti et al. (47), no study has investigated the use of different types of cannabis in patients with a psychotic disorder. The extent to which the presence or absence of CBD in cannabis will influence the early occurrence of a first-episode psychosis or to what extent it will affect the course of the disease is, therefore, unknown.

Anxiety and panic attacks are the most commonly reported adverse reactions following the use of cannabis. Inexperience and use in a foreign environment play a major role (58). Though anxiety and panic attacks are often reported, many users take cannabis for its fear-inhibiting effects [for a review, see Ref. (59)]. THC seems to be responsible for the anxiogenic effects of cannabis [e.g., Ref. (58, 60, 61)].

By the early 1980s, it had been shown that THC led to a significant increase of acute anxiety symptoms, while CBD had no effect (42). When CBD and THC were administered together, the anxiogenic effect of THC was halved. This was an important indication that the anxiety-inducing effects of THC could be antagonized by CBD. The results from later studies, however, were inconsistent; the anxiety-reducing effect of CBD was not found in all subsequent studies. Ilan and colleagues investigated the contribution of THC and CBD to the subjective and behavioral effects of smoked marijuana (62). In their study, 23 healthy marijuana users were randomly assigned to a low- or a high-THC group and low or high levels of CBD. In the four sessions under blinded conditions, subjects smoked marijuana cigarettes containing placebo (no active cannabinoids) or cigarettes containing THC with low or high levels of CBD. Compared with the placebo, cannabis caused a slight short-term increase in anxiety symptoms (VAS). These effects were greatest in the high-THC condition and appeared to diminish when the CBD content was high, but this latter effect was not statistically significant. Because this increase in anxiety was generally mild and because not all subjects responded with fear, a follow-up analysis with only the anxious subjects was performed. There was a non-significant trend for less anxiety in the high- versus the low-CBD condition in subjects who reported higher levels of anxiety after smoking the joints. A reason for the absence of significant results in this study might be that neither the THC nor the CBD concentrations were high enough to have significant effects. In the studies in which anxiety-reducing effects were reported, high oral doses of CBD typically were involved. Cannabis that is used for recreational purposes does not contain such high amounts of CBD.

People with cannabis dependence are more likely to suffer from an anxiety disorder and, in particular, from social anxiety disorder [for a review, see Ref. (58)].

So far, studies investigating the relationship between cannabis dependence and anxiety disorders have not clarified the nature of the relationship in question: does cannabis use lead to anxiety disorders or do anxiety disorders lead to the (over-) use of cannabis? There are no studies in which the relationship between cannabis use and anxiety disorders is examined and in which an inquiry about the type of cannabis used or its THC/CBD ratio is included.

In two experiments using patients suffering from social anxiety disorder along with healthy volunteers as controls, the subjects had to speak in front of a video camera, regardless of whether they were under the influence of CBD. In this experimental situation, CBD was effective in preventing symptoms of anxiety, both in healthy volunteers and in patients with social anxiety disorder (41, 63). CBD suppressed the symptoms of anxiety, similar to the action of the sedatives diazepam and ipsapirone. The main features of the studies on humans that have investigated the psychological effects of administering CBD (singularly or in combination with THC) are summarized in Table 2.

**Table 2:** **Overview of studies investigating the effect of cannabidiol or cannabidiol in combination with THC on psychological functions in humans. Studies in which cannabis extracts have been used are not included**.

Reference	Subjects	Dosing THC/CBD	Results	Comments
Karniol et al. (64)	Healthy volunteers (*n* = 40)	30 mg THC (oral); 15, 30 of 60 mg CBD (oral) or in combination with 30 mg THC (both oral)	Antagonizing (part of) the THC-induced effects	CBD decreased the anxiety component of THC effects; no effect of CBD alone
Hollister and Gillespie (65)	Healthy volunteers (*n* = 30)	20 mg THC + 40 mg CBD (both oral)	CBD delays onset of the effect of THC and prolongs the effects of THC	
Dalton et al. (66)	Healthy volunteers (*n* = 15)	25 μg/kg BW THC and 150 μg/kg BW CBD via smoking a joint	CBD reduces euphoric effect of THC	Only effective when CBD and THC are administered simultaneously
Hollister (67)	Healthy volunteers (*n* = ?)	CBD 5–30 mg i.v.	No effects	
Carlini and Cunha (68)	Healthy volunteers	Acute 600 mg CBD; 10 mg/kg/BW CBD 20 days	CBD does not have psychological or physical effects	Light drowsiness after CBD administration
Zuardi et al. (42)	Healthy volunteers (*n* = 8)	0.5 mg/kg BW THC + 1 mg/kg BW CBD (both oral)	CBD antagonizes psychological effects of THC (anxiety)	CBD itself has no effect and does not antagonize the physical effects of THC (HR, BP)
Zuardi et al. (69)	Treatment resistant schizophrenic patients (*n* = 3)	CBD during 29 days upwards from 40 to 1280 mg/day (oral)	CBD does not antagonize symptoms	No side effects of CBD reported
Crippa et al. (70)	Healthy volunteers (*n* = 10)	CBD 400 mg oral	Anxiolytic effects; light mental sedation	SPECT results: effects in left amygdala-hippocampus complex radiating to hypothalamus
Leweke et al. (71)	Psychiatric patients (*n* = 43)	CBD oral 800 mg/day; during 4 weeks	CBD more effective as antipychotic than amsulpride	Less side effects of CBD than with amsulpride
Zuardi et al. (72)	PD patients with psychoses	CBD 150 mg/day; during 4 weeks	CBD possibly effective for treatment of PD patients suffering from psychoses	No significant side effects of CBD reported
Borgwardt et al. (73), Fusar-Poli et al. (74), Fusar-Poli et al. (75), Bhattacharyya et al. (76)^a^	Healthy volunteers (*n* = 15)	CBD oral 600 mg; 10 mg THC (not simultaneously); in comparison with placebo	No effect in contrast with THC; CBD activates other brain areas than THC no effects of CBD in verbal learning task and no induction of psychotic symptoms	No sedation and no inhibition of locomotion by CBD; THC induces psychotic symptoms, anxiety, and sedation
Zuardi et al. (77)	Patients with bipolar disorder (*n* = 2)	CBD oral 600 – 1200 mg/day during 25 days	CBD has no effect on symptoms	No side effects of CBD reported
Bhattacharyya et al. (43)	Healthy volunteers (*n* = 6)	CBD 5 mg i.v. immediately followed by 1.25 mg THC i.v.	CBD antagonizes THC-induced psychotic symptoms	CBD and THC have opposite effects on regional brain function
Bergamaschi et al. (78)	Healthy controls (*n* = 12) and patients with social phobia (*n* = 24)	CBD oral 600 mg	Reduction of anxiety scores in patients, no effect in controls	No physical effects or side effects of CBD reported
Crippa et al. (79)	Patients with social phobia (*n* = 10)	CBD oral 400 mg	No effect on psychological scores	No physical effects; SPECT: CBD exerts its effects via limbic and paralimbic areas
Nicholson et al. (80)	Healthy volunteers (*n* = 8)	CBD 5 mg + THC 5 mg; CBD 15 mg + THC 15 mg, via mouth spray	THC (15 mg) increases drowsiness, antagonized by CBD (15 mg)	
Hallak et al. (81)	Schizophrenic patients (*n* = 28)	CBD oral 300 and 600 mg acute	No positive effects in Stroop Color Word Test	No significant side effects of CBD reported
Hallak et al. (82)	Healthy volunteers (*n* = 10)	CBD oral 600 mg and ketamine i.v.	CBD increases activating effects of ketamine (BPRS); reduction of ketamine-induced depersonalization (CADSS)	No effect of CBD on HR and BP

^a^ This concerns experiments with one group of 15 subjects from which the results have been spread over four different publications; BP, blood pressure; BW, body weight; CADSS, Clinician Administered Dissociative States Scale; HR, heart rate; i.v., intravenously; PD, Parkinson disease.

Several studies have shown that cannabis and THC dose-dependently cause cognitive and psychomotor function impairments along with memory, (selective) attention, locomotion, perception, and response impairments (83–85). The effects occur most strongly during the first hour after smoking a joint and between 1 and 2 h after oral intake. Little experimental research exists on the effects of CBD alone or in conjunction with THC on cognitive and psychomotor functions. The studies performed so far show few “protective” effects of CBD on cognitive functions. Morgan and colleagues identified a few such effects on memory functions, but the research on this aspect of CBD has inconsistent findings (45, 49).

Although no human studies have specifically investigated the long-term effects of the combined effect of THC and CBD on cognitive functioning, there are indications that CBD may have some neuroprotective properties. In some neurodegenerative diseases that are often associated with declines in cognitive functioning, such as Alzheimer’s and Parkinson’s diseases, CBD may have some role in treatment or prevention (86–89).

The ratio of THC to CBD may play a role in the risk of addiction (90). Morgan and colleagues examined whether there is a difference in attentional bias between users of cannabis having a relatively high CBD/THC ratio versus cannabis having a low-CBD/THC ratio. Much weaker attentional bias for cannabis-related stimuli was found for users of cannabis with a high CBD content than for users of cannabis with a low-CBD content. Furthermore, the extent to which both groups appreciated the self-selected drug and the strength of the desire for their drug (“wanting”) were investigated. High CBD content led to diminished appreciation and weaker desire for the drug relative to low-CBD content. The researchers concluded that cannabis with a high CBD content confers less risk for developing an addiction than cannabis with a low-CBD content (90). Whether smoking hashish in practice diminishes addiction risk in comparison with smoking highly potent marijuana should be further investigated.

## Conclusion

Cannabis is not a safe drug. Depending on how often someone uses, the age of onset, the potency of the cannabis that is used and someone’s individual sensitivity, the recreational use of cannabis may cause permanent psychological disorders. Many recreational users of cannabis will never be faced with serious or permanent health deficits. However, for some users, the use of cannabis may cause undesirable psychological side effects, such as cognitive impairment, anxiety and paranoia, and an increased risk of developing chronic psychosis and addiction. Despite all of the publicity surrounding cannabis, remarkably few studies have been performed that examined the relationship between a possibly harmful effect of THC and a possibly protective effect of CBD. The few studies that exist on the effects of CBD show that this cannabinoid can counteract some of the negative effects of THC, although their results have not always been consistent. The question remains how the findings from laboratory studies, often employing high doses of CBD and high CBD/THC ratios, can be extrapolated to the typical practices of the recreational cannabis user. Few or no adverse effects of CBD have been proffered, and where CBD has been found to have an effect, it is usually in a “positive” (i.e., salubrious) direction. The evidence favoring a beneficial effect of CBD therefore merits further investigation in studies in which the amounts and ratios of CBD and THC correspond to the daily practices of recreational cannabis use.

## Conflict of Interest Statement

The authors declare that the research was conducted in the absence of any commercial or financial relationships that could be construed as a potential conflict of interest.

## References

[1] Howlett AC Johnson MR Melvin LS Milne GM Nonclassical cannabinoid analgetics inhibit adenylate cyclase: development of a cannabinoid receptor model. Mol Pharmacol (1988) 33(3):297–302 3352594

[2] Matsuda LA Lolait SJ Brownstein MJ Young AC Bonner TI Structure of a cannabinoid receptor and functional expression of the cloned cDNA. Nature (1990) 346(6284):561–4 10.1038/346561a0 2165569

[3] Munro S Thomas KL Bu-Shaar M Molecular characterization of a peripheral receptor for cannabinoids. Nature (1993) 365(6441):61–5 10.1038/365061a0 7689702

[4] Herkenham M Lynn AB Little MD Johnson MR Melvin LS De Costa BR Cannabinoid receptor localization in brain. Proc Natl Acad Sci U S A (1990) 87(5):1932–6 10.1073/pnas.87.5.1932 2308954 PMC53598

[5] Glass M Dragunow M Faull RL Cannabinoid receptors in the human brain: a detailed anatomical and quantitative autoradiographic study in the fetal, neonatal and adult human brain. Neuroscience (1997) 77(2):299–318 10.1016/S0306-4522(96)00428-9 9472392

[6] Devane WA Hanus L Breuer A Pertwee RG Stevenson LA Griffin G Isolation and structure of a brain constituent that binds to the cannabinoid receptor. Science (1992) 258(5090):1946–9 10.1126/science.1470919 1470919

[7] Sugiura T Kondo S Sukagawa A Nakane S Shinoda A Itoh K 2-Arachidonoylglycerol: a possible endogenous cannabinoid receptor ligand in brain. Biochem Biophys Res Commun (1995) 215(1):89–97 10.1006/bbrc.1995.2437 7575630

[8] Stella N Schweitzer P Piomelli D A second endogenous cannabinoid that modulates long-term potentiation. Nature (1997) 388(6644):773–8 10.1038/42015 9285589

[9] Wilson RI Nicoll RA Endocannabinoid signaling in the brain. Science (2002) 296(5568):678–82 10.1126/science.1063545 11976437

[10] Grotenhermen F Pharmacology of cannabinoids. Neuro Endocrinol Lett (2004) 25(1-2):14–23 15159677

[11] Skaper SD Di Marzo V Endocannabinoids in nervous system health and disease: the big picture in a nutshell. Philos Trans R Soc Lond B Biol Sci (2012) 367(1607):3193–200 10.1098/rstb.2012.0313 23108539 PMC3481537

[12] Alger BE Kim J Supply and demand for endocannabinoids. Trends Neurosci (2011) 34(6):304–15 10.1016/j.tins.2011.03.003 21507493 PMC3106144

[13] Di Marzo V The endocannabinoid system: its general strategy of action, tools for its pharmacological manipulation and potential therapeutic exploitation. Pharmacol Res (2009) 60(2):77–84 10.1016/j.phrs.2009.02.010 19559360

[14] Fisar Z Phytocannabinoids and endocannabinoids. Curr Drug Abuse Rev (2009) 2(1):51–75 10.2174/1874473710902010051 19630737

[15] Hill AJ Williams CM Whalley BJ Stephens GJ Phytocannabinoids as novel therapeutic agents in CNS disorders. Pharmacol Ther (2012) 133:79–97 10.1016/j.pharmthera.2011.09.002 21924288

[16] Izzo AA Borrelli F Capasso R Di Marzo V Mechoulam R Non-psychotropic plant cannabinoids: new therapeutic opportunities from an ancient herb. Trends Pharmacol Sci (2009) 30:515–27 10.1016/j.tips.2009.07.006 19729208

[17] Ligresti A Cascio MG Pryce G Kulasegram S Beletskaya I De Petrocellis L New potent and selective inhibitors of anandamide reuptake with antispastic activity in a mouse model of multiple sclerosis. Br J Pharmacol (2006) 147(1):83–91 10.1038/sj.bjp.0706418 16284631 PMC1615845

[18] Bisogno T Maccarrone M De Petrocellis L Jarrahian A Finazzi-Agro A Hillard C The uptake by cells of 2-arachidonoylglycerol, an endogenous agonist of cannabinoid receptors. Eur J Biochem (2001) 268(7):1982–9 10.1046/j.1432-1327.2001.02072.x 11277920

[19] Russo EB Burnett A Hall B Parker KK Agonistic properties of cannabidiol at 5-HT1a receptors. Neurochem Res (2005) 30(8):1037–43 10.1007/s11064-005-6978-1 16258853

[20] Campos AC Guimarães FS Involvement of 5HT1A receptors in the anxiolytic-like effects of cannabidiol injected into the dorsolateral periaqueductal gray of rats. Psychopharmacology (Berl) (2008) 199(2):223–30 10.1007/s00213-008-1168-x 18446323

[21] Demirakca T Sartorius A Ende G Meyer N Welzel H Skopp G Diminished gray matter in the hippocampus of cannabis users: possible protective effects of cannabidiol. Drug Alcohol Depend (2011) 114(2-3):242–5 10.1016/j.drugalcdep.2010.09.020 21050680

[22] Zuardi AW Hallak JE Crippa JA Interaction between cannabidiol (CBD) and (9)-tetrahydrocannabinol (THC): influence of administration interval and dose ratio between the cannabinoids. Psychopharmacology (Berl) (2012) 219(1):247–9 10.1007/s00213-011-2495-x 21947314

[23] Arnold JC Boucher AA Karl T The yin and yang of cannabis-induced psychosis: the actions of delta(9)-tetrahydrocannabinol and cannabidiol in rodent models of schizophrenia. Curr Pharm Des (2012) 8(32):5113–30 10.2174/138161212802884726 22716133

[24] Fischedick J van der Kooy F Verpoorte R Cannabinoid receptor 1 binding activity and quantitative analysis of *Cannabis sativa L*. smoke and vapor. Chem Pharm Bull (Tokyo) (2010) 58(2):201–7 10.1248/cpb.58.201 20118579

[25] Zuurman L Roy C Schoemaker RC Hazekamp A den Hartigh J Bender JC Effect of intrapulmonary tetrahydrocannabinol administration in humans. J Psychopharmacol (2008) 22(7):707–16 10.1177/0269881108089581 18515447

[26] Harvey DJ Samaram E Mechoulam R Comparative metabolism of cannabidiol in dog, rat and man. Pharmacol Biochem Behav (1991) 40(3):523–32 10.1016/0091-3057(91)90358-9 1806942

[27] Bergamaschi MM Queiroz RH Zuardi AW Crippa JA Safety and side effects of cannabidiol, a *Cannabis sativa* constituent. Curr Drug Saf (2011) 6(4):237–49 10.2174/157488611798280924 22129319

[28] Reece AS Chronic toxicology of cannabis. Clin Toxicol (Phila) (2009) 47(6):517–24 10.1080/15563650903074507 19586351

[29] Lorenzetti V Lubman DI Whittle S Solowij N Yucel M Structural MRI findings in long-term cannabis users: what do we know? Subst Use Misuse (2010) 45(11):1787–808 10.3109/10826084.2010.482443 20590400

[30] Hermann D Schneider M Potential protective effects of cannabidiol on neuroanatomical alterations in cannabis users and psychosis: a critical review. Curr Pharm Des (2012) 18(32):4897–905 10.2174/138161212802884825 22716143

[31] Breivogel CS Childers SR The functional neuroanatomy of brain cannabinoid receptors. Neurobiol Dis (1998) 5(6 Pt B):417–31 10.1006/nbdi.1998.0229 9974175

[32] Ameri A The effects of cannabinoids on the brain. Prog Neurobiol (1999) 58:315–48 10.1016/S0301-0082(98)00087-2 10368032

[33] Iversen L Cannabis and the brain. Brain (2003) 126(Pt 6):1252–70 10.1093/brain/awg143 12764049

[34] Matochik JA Eldreth DA Cadet JL Bolla KI Altered brain tissue composition in heavy marijuana users. Drug Alcohol Depend (2005) 77(1):23–30 10.1016/j.drugalcdep.2004.06.011 15607838

[35] Yucel M Zalesky A Takagi MJ Bora E Fornito A Ditchfield M White-matter abnormalities in adolescents with long-term inhalant and cannabis use: a diffusion magnetic resonance imaging study. J Psychiatry Neurosci (2010) 35(6):409–12 10.1503/jpn.090177 20731960 PMC2964371

[36] Wilson W Mathew R Turkington T Hawk T Coleman RE Provenzale J Brain morphological changes and early marijuana use: a magnetic resonance and positron emission tomography study. J Addict Dis (2000) 19(1):1–22 10.1300/J069v19n01_01 10772599

[37] D’Souza DC Cannabinoids and psychosis. Int Rev Neurobiol (2007) 2007(78):289–326 10.1016/S0074-7742(06)78010-2 17349865

[38] D’Souza DC Sewell RA Ranganathan M Cannabis and psychosis/schizophrenia: human studies. Eur Arch Psychiatry Clin Neurosci (2009) 259(7):413–31 10.1007/s00406-009-0024-2 19609589 PMC2864503

[39] Barkus E Murray RM Substance use in adolescence and psychosis: clarifying the relationship. Annu Rev Clin Psychol (2010) 2010(6):365–89 10.1146/annurev.clinpsy.121208.131220 20192802

[40] Rottanburg D Robins AH Ben-Arie O Teggin A Elk R Cannabis-associated psychosis with hypomanic features. Lancet (1982) 2(8312):1364–6 10.1016/S0140-6736(82)91270-3 6129463

[41] Zuardi AW Cannabidiol: from an inactive cannabinoid to a drug with wide spectrum of action. Rev Bras Psiquiatr (2008) 30(3):271–80 10.1590/S1516-44462008000300015 18833429

[42] Zuardi AW Shirakawa I Finkelfarb E Karniol IG Action of cannabidiol on the anxiety and other effects produced by delta 9-THC in normal subjects. Psychopharmacology (Berl) (1982) 76(3):245–50 10.1007/BF00432554 6285406

[43] Bhattacharyya S Morrison PD Fusar-Poli P Martin-Santos R Borgwardt S Winton-Brown T Opposite effects of delta-9-tetrahydrocannabinol and cannabidiol on human brain function and psychopathology. Neuropsychopharmacology (2010) 35(3):764–74 10.1038/npp.2009.184 19924114 PMC3055598

[44] Schubart CD van Gastel WA Breetvelt EJ Beetz SL Ophoff RA Sommer IE Cannabis use at a young age is associated with psychotic experiences. Psychol Med (2010) 7:1–10 10.1017/S003329171000187X 20925969

[45] Morgan CJ Curran HV Effects of cannabidiol on schizophrenia-like symptoms in people who use cannabis. Br J Psychiatry (2008) 192(4):306–7 10.1192/bjp.bp.107.046649 18378995

[46] Morgan CJ Schafer G Freeman TP Curran HV Impact of cannabidiol on the acute memory and psychotomimetic effects of smoked cannabis: naturalistic study [corrected]. Br J Psychiatry (2010) 197(4):285–90 10.1192/bjp.bp.110.077503 20884951

[47] Di Forti M Morgan C Dazzan P Pariante C Mondelli V Marques TR High-potency cannabis and the risk of psychosis. Br J Psychiatry (2009) 195(6):488–91 10.1192/bjp.bp.109.064220 19949195 PMC2801827

[48] Schubart CD Sommer IE van Gastel WA Goetgebuer RL Kahn RS Boks MP Cannabis with high cannabidiol content is associated with fewer psychotic experiences. Schizophr Res (2011) 130(1–3):216–21 10.1016/j.schres.2011.04.017 21592732

[49] Morgan CJ Gardener C Schafer G Swan S Demarchi C Freeman TP Sub-chronic impact of cannabinoids in street cannabis on cognition, psychotic-like symptoms and psychological well-being. Psychol Med (2011) 29:1–10 21798112 10.1017/S0033291711001322

[50] Moore TH Zammit S Lingford-Hughes A Barnes TR Jones PB Burke M Cannabis use and risk of psychotic or affective mental health outcomes: a systematic review. Lancet (2007) 370:319–28 10.1016/S0140-6736(07)61162-3 17662880

[51] Kuepper R van Os J Lieb R Wittchen HU Höfler M Henquet C Continued cannabis use and risk of incidence and persistence of psychotic symptoms: 10 year follow-up cohort study. BMJ (2011) 342:d738 10.1136/bmj.d738 21363868 PMC3047001

[52] Griffith-Lendering MF Wigman JT Prince van Leeuwen A Huijbregts SC Huizink AC Ormel J Cannabis use and vulnerability for psychosis in early adolescence – a TRAILS study. Addiction (2013) 108(4):733–40 10.1111/add.12050 23216690

[53] Dragt S Nieman DH Schultze-Lutter F van der Meer F Becker H de Haan L Cannabis use and age at onset of symptoms in subjects at clinical high risk for psychosis. Acta Psychiatr Scand (2012) 125(1):45–53 10.1111/j.1600-0447.2011.01763.x 21883099

[54] Large M Sharma S Compton MT Slade T Nielssen O Cannabis use and earlier onset of psychosis: a systematic meta-analysis. Arch Gen Psychiatry (2011) 68(6):555–61 10.1001/archgenpsychiatry.2011.5 21300939

[55] Degenhardt L Roxburgh A McKetin R Hospital separations for cannabis- and methamphetamine-related psychotic episodes in Australia. Med J Aust (2007) 186(7):342–5 17407429 10.5694/j.1326-5377.2007.tb00933.x

[56] Caspari D Cannabis and schizophrenia: results of a follow-up study. Eur Arch Psychiatry Clin Neurosci (1999) 249(1):45–9 10.1007/s004060050064 10195343

[57] Linszen DH Dingemans PM Lenior ME Cannabis abuse and the course of recent-onset schizophrenic disorders. Arch Gen Psychiatry (1994) 51(4):273–9 10.1001/archpsyc.1994.03950040017002 8161287

[58] Crippa JA Zuardi AW Martin-Santos R Bhattacharyya S Atakan Z McGuire P Cannabis and anxiety: a critical review of the evidence. Hum Psychopharmacol (2009) 24(7):515–23 10.1002/hup.1048 19693792

[59] Moreira FA Wotjak CT Cannabinoids and anxiety. Curr Top Behav Neurosci (2010) 2:429–50 10.1007/7854_2009_16 21309120

[60] Tambaro S Bortolato M Cannabinoid-related agents in the treatment of anxiety disorders: current knowledge and future perspectives. Recent Pat CNS Drug Discov (2012) 7(1):25–40 10.2174/157488912798842269 22280339 PMC3691841

[61] Karschner EL Darwin WD McMahon RP Liu F Wright S Goodwin RS Subjective and physiological effects after controlled Sativex and oral THC administration. Clin Pharmacol Ther (2011) 89(3):400–7 10.1038/clpt.2010.318 21289620 PMC3836266

[62] Ilan AB Gevins A Coleman M ElSohly MA de Wit H Neurophysiological and subjective profile of marijuana with varying concentrations of cannabinoids. Behav Pharmacol (2005) 16:487–96 10.1097/00008877-200509000-00023 16148455

[63] Zuardi AW Cosme RA Graeff FG Guimaraes FS Effects of ipsapirone and cannabidiol on human experimental anxiety. J Psychopharmacol (1993) 7(1 Suppl):82–8 10.1177/026988119300700112 22290374

[64] Karniol IG Shirakawa I Kasinski N Pfeferman A Carlini EA Cannabidiol interferes with the effects of delta 9-tetrahydrocannabinol in man. Eur J Pharmacol (1974) 28:172–7 10.1016/0014-2999(74)90129-0 4609777

[65] Hollister LE Gillespie H Interactions in man of delta-9-tetrahydrocannabinol. II. Cannabinol and cannabidiol. Clin Pharmacol Ther (1975) 18:80–3 1097148 10.1002/cpt197518180

[66] Dalton WS Martz R Lemberger L Rodda BE Forney RB Influence of cannabidiol on delta-9-tetrahydrocannabinol effects. Clin Pharmacol Ther (1976) 19:300–9 770048 10.1002/cpt1976193300

[67] Hollister LE Cannabidiol and cannabinol in man. Experientia (1973) 29:825–6 10.1007/BF01946311 4724713

[68] Carlini EA Cunha JM Hypnotic and antiepileptic effects of cannabidiol. J Clin Pharmacol (1981) 21:417S–27 10.1002/j.1552-4604.1981.tb02622.x 7028792

[69] Zuardi AW Hallak JE Dursun SM Morais SL Sanches RF Musty RE Cannabidiol monotherapy for treatment-resistant schizophrenia. J Psychopharmacol (2006) 20:683–6 10.1177/0269881106060967 16401651

[70] Crippa JA Zuardi AW Garrido GE Wichert-Ana L Guarnieri R Ferrari L Effects of cannabidiol (CBD) on regional cerebral blood flow. Neuropsychopharmacology (2004) 29:417–26 10.1038/sj.npp.1300340 14583744

[71] Leweke FM Koethe D Pahlisch F Schreiber D Gert CW Nolden BM Antipsychotic effects of cannabidiol. European Psychiatry (2009) 24:S207

[72] Zuardi AW Crippa JA Hallak JE Pinto JP Chagas MH Rodrigues GG Cannabidiol for the treatment of psychosis in Parkinson’s disease. J Psychopharmacol (2009) 23:979–83 10.1177/0269881108096519 18801821

[73] Borgwardt SJ Allen P Bhattacharyya S Fusar-Poli P Crippa JA Seal ML Neural basis of delta-9-tetrahydrocannabinol and cannabidiol: effects during response inhibition. Biol Psychiatry (2008) 64:966–73 10.1016/j.biopsych.2008.05.011 18589404

[74] Fusar-Poli P Crippa JA Bhattacharyya S Borgwardt SJ Allen P Martin-Santos R Distinct effects of (delta)9-tetrahydrocannabinol and cannabidiol on neural activation during emotional processing. Arch Gen Psychiatry (2009) 66:95–105 10.1001/archgenpsychiatry.2008.519 19124693

[75] Fusar-Poli P Allen P Bhattacharyya S Crippa JA Mechelli A Borgwardt S Modulation of effective connectivity during emotional processing by delta 9-tetrahydrocannabinol and cannabidiol. Int J Neuropsychopharmacol (2010) 13: 421–32 10.1017/S1461145709990617 19775500

[76] Bhattacharyya S Fusar-Poli P Borgwardt S Martin-Santos R Nosarti C O’Carroll C Modulation of mediotemporal and ventrostriatal function in humans by Delta9-tetrahydrocannabinol: a neural basis for the effects of Cannabis sativa on learning and psychosis. Arch Gen Psychiatry (2009) 66(4):442–51 10.1001/archgenpsychiatry.2009.17 19349314

[77] Zuardi A Crippa J Dursun S Morais S Vilela J Sanches R Cannabidiol was ineffective for manic episode of bipolar affective disorder. J Psychopharmacol (2010) 24:135–7 10.1177/0269881108096521 18801823

[78] Bergamaschi MM Queiroz RH Chagas MH de Oliveira DC De Martinis BS Kapczinski F Cannabidiol reduces the anxiety induced by simulated public speaking in treatment-naive social phobia patients. Neuropsychopharmacology (2011) 36:1219–26 10.1038/npp.2011.6 21307846 PMC3079847

[79] Crippa JA Derenusson GN Ferrari TB Wichert-Ana L Duran FL Martin-Santos R Neural basis of anxiolytic effects of cannabidiol (CBD) in generalized social anxiety disorder: a preliminary report. J Psychopharmacol (2011) 25:121–30 10.1177/0269881110379283 20829306

[80] Nicholson AN Turner C Stone BM Robson PJ Effect of delta-9-tetrahydrocannabinol and cannabidiol on nocturnal sleep and early-morning behavior in young adults. J Clin Psychopharmacol (2004) 24:305–13 10.1097/01.jcp.0000125688.05091.8f 15118485

[81] Hallak JE Hado-de-Sousa JP Crippa JA Sanches RF Trzesniak C Chaves C Performance of schizophrenic patients in the Stroop Color Word Test and electrodermal responsiveness after acute administration of cannabidiol (CBD). Rev Bras Psiquiatr (2010) 32:56–61 10.1590/S1516-44462010000100011 20339735

[82] Hallak JE Dursun SM Bosi DC de Macedo LR Hado-de-Sousa JP Abrao J The interplay of cannabinoid and NMDA glutamate receptor systems in humans: preliminary evidence of interactive effects of cannabidiol and ketamine in healthy human subjects. Prog Neuropsychopharmacol Biol Psychiatry (2011) 35:198–202 10.1016/j.pnpbp.2010.11.002 21062637

[83] Ramaekers JG Berghaus G van Laar M Drummer OH Dose related risk of motor vehicle crashes after cannabis use. Drug Alcohol Depend (2004) 73(2):109–19 10.1016/j.drugalcdep.2003.10.008 14725950

[84] Ranganathan M D’Souza DC The acute effects of cannabinoids on memory in humans: a review. Psychopharmacology (Berl) (2006) 188(4):425–44 10.1007/s00213-006-0508-y 17019571

[85] Hunault CC Mensinga TT Böcker KB Schipper CM Kruidenier M Leenders ME Cognitive and psychomotor effects in males after smoking a combination of tobacco and cannabis containing up to 69 mg delta-9-tetrahydrocannabinol (THC). *Psychopharmacology (Berl)* (2009) 204(1):85–94 10.1007/s00213-008-1440-0 19099294

[86] Hampson AJ Grimaldi M Lolic M Wink D Rosenthal R Axelrod J Neuroprotective antioxidants from marijuana. Ann N Y Acad Sci (2000) 899:274–82 10.1111/j.1749-6632.2000.tb06193.x 10863546

[87] Lastres-Becker I Molina-Holgado F Ramos JA Mechoulam R Fernandez-Ruiz J Cannabinoids provide neuroprotection against 6-hydroxydopamine toxicity in vivo and in vitro: relevance to Parkinson’s disease. Neurobiol Dis (2005) 19(1–2):96–107 10.1016/j.nbd.2004.11.009 15837565

[88] Harvey BS Ohlsson KS Maag JL Musgrave IF Smid SD Contrasting protective effects of cannabinoids against oxidative stress and amyloid-beta evoked neurotoxicity in vitro. Neurotoxicology (2012) 33(1):138–46 10.1016/j.neuro.2011.12.015 22233683

[89] Scuderi C Filippis DD Iuvone T Blasio A Steardo A Esposito G Cannabidiol in medicine: a review of its therapeutic potential in CNS disorders. Phytother Res (2009) 23(5):597–602 10.1002/ptr.2625 18844286

[90] Morgan CJ Freeman TP Schafer GL Curran HV Cannabidiol attenuates the appetitive effects of delta 9-tetrahydrocannabinol in humans smoking their chosen cannabis. Neuropsychopharmacology (2010) 35(9):1879–85 10.1038/npp.2010.58 20428110 PMC2906701

